# Kinetic and kinematic differences between deadlifts and goodmornings

**DOI:** 10.1186/2052-1847-5-27

**Published:** 2013-12-07

**Authors:** Florian Schellenberg, Julia Lindorfer, Renate List, William R Taylor, Silvio Lorenzetti

**Affiliations:** 1 Institute for Biomechanics, ETH Zurich, HCI E 351, Wolfgang-Pauli-Strasse 10, 8093 Zürich, Switzerland; 2 Department of Sports Engineering & Biomechanics, University of Applied Sciences Technikum Wien, Höchstädtplatz 6, 1200 Wien, Austria

**Keywords:** Trunk movement, Spine, Motion analysis, Loading conditions, Free weights

## Abstract

**Background:**

In order to improve training performance, as well as avoid overloading during prevention and rehabilitation exercises in patients, the aim of this study was to understand the biomechanical differences in the knee, hip and the back between the exercises “Goodmornings” (GMs) and “Deadlifts” (DLs).

**Methods:**

The kinetics and kinematics of 13 subjects, performing GMs and DLs with an additional 25% (GMs), 25% and 50% (DLs) body weight (BW) on the barbell were analysed. Using the kinetic and kinematic data captured using a 3D motion analysis and force plates, an inverse approach with a quasi-static solution was used to calculate the sagittal moments and angles in the knee, hip and the trunk. The maximum moments and joint angles were statistically tested using ANOVA with a Bonferroni adjustment.

**Results:**

The observed maximal flexion angle of the knee was 5.3 ± 6.7° for GMs and 107.8 ± 22.4° and 103.4 ± 22.6° for DLs with 25% and 50% BW respectively. Of the hip, the maximal flexion angle was 25% smaller during GMs compared to DLs. No difference in kinematics of the trunk between the two exercises was observed. For DLs, the resulting sagittal moment in the knee was an external flexion moment, whereas during GMs an external extension moment was present. Importantly, no larger sagittal knee joint moments were observed when using a heavier weight on the barbell during DLs, but higher sagittal moments were found at the hip and L4/L5. Compared to GMs, DLs produced a lower sagittal moment at the hip using 25% BW while generating the same sagittal moment at L4/L5.

**Conclusions:**

The two exercises exhibited different motion patterns for the lower extremities but not for the trunk. To strengthen the hip while including a large range of motion, DLs using 50% BW should be chosen. Due to their ability to avoid knee flexion or a knee flexion moment, GMs should be preferentially chosen over DLs as ACL rupture prevention exercises. Here, in order to shift the hamstring to quadriceps ratio towards the hamstrings, GMs should be favoured ahead of DLs using 50% BW before DLs using 25% BW.

## Background

Strength training exercises such as Deadlifts (DLs) or Goodmornings (GMs) are commonly used in prevention programs for reducing the risk of ACL injury or for rehabilitating low back pain patients, as well as during training to increase an athlete’s specific performance, where the loading conditions play an important role on both the passive and active musculoskeletal structures. Here, exercise kinematics play a key role for governing the lifting mechanics, and therefore modulating the risk of injury and level of performance [1]. In 1999 in Switzerland, the most frequent injuries during fitness training were the shoulder 24.4%, back 16.6%, thigh 11.0% and knee 8.8% [2]. The reasons for injury were predominantly attributed to overloading (45.6%) and wrong execution of the exercise (21.1%) [2]. Despite these statistics, a complete biomechanical understanding of the loading conditions of many exercises during strength training remains lacking.

The DL is a multi-joint resistance exercise that is performed in a variety of training settings [3]. It begins with the lifter in a squat position, with arms straight and pointing downwards, with an alternating hand grip on the bar [1]. The movement includes mainly an extension of the knee and hip until the body reaches an upright standing position. The lifting exercise then uses the following muscles: *gluteus maximus*, *erector spinae*, hamstrings, quadriceps, *trapezius*, *rhomboideus*, *deltoideus* and finger flexors [4]. Due to the fact that the DL is a closed chain exercise [5], it is often used in the prevention of and rehabilitation after anterior cruciate ligament (ACL) reconstruction to improve strength of the muscular structures that surround the knee and hence dynamic stability of the joint [6-8]. The DL is also one of the three disciplines in powerlifting. The biomechanics of the lift have been studied extensively during competition, focusing on the sumo and conventional styles [1,6,9], where the maximal isometric forces in four different positions during DLs were shown to result in a higher potential to increase the force toward the end of the DL (from 3380 to 5829 N) [10]. Training using these exercises has also been clearly related to functional adaptation of the spine, where the annual lifted loads of power-lifters has been shown to correlate with the bone mineral content in L3 [11]. However, the increased forward trunk tilt during DL lift-off may predispose the spine and back musculature to an increased risk of injury [11,12]. In response to this, Cholewicki and co-workers [12] demonstrated that a more upright trunk at lift-off is able to reduce anterior shear force at the lumbar L4/L5 joint. Furthermore, Escamilla and co-workers [1] showed the importance of keeping the barbell mass as close as possible to the body in order to minimise injury risk to the back as well as to enhance performance. No statistically significant differences were found in this study between the kinematics of high and low-skilled lifters, but they did show differences regarding how the barbell passed the knee: Highly skilled lifters kept the barbell mass closer to the body than less-skilled lifters.

The GM exercise is an assistance movement utilized primarily by weight lifters to strengthen the extensors of the torso, the *gluteus*, hamstrings and *erector spinae*[13]. Starting in an upright standing position and with the barbell on the shoulders, the hips are progressively flexed until maximum hip flexion is reached, but the knees remained straight throughout. GMs are a good exercise for specifically conditioning lumbar-thoracic flexion and extension of the back [14], but for all level of performance, good lifting technique is required when approaching near maximal effort to avoid acute injury or long-term damage. Here, the low back must have sufficient strength to keep the body in the correct position, since high *erector spinae* forces are known to occur, resulting in high shear and compressive forces at the level of L5/S1 [15]. The authors stated the importance of sufficiently conditioned lower back musculature and proper sport technique for reducing the risk of back injury [15,16]. In the review of Carpenter DM and Nelson BW [17], the recommendation for low back pain patients was to train using lumbar extension reconditioning exercises with the pelvis stabilized in a specific, progressive and intensive manner, since this was shown to lead to the most favourable improvements in low back strength, muscle cross-sectional area and vertebral bone mineral density. The latter recommendation is in agreement with the general finding that strength training is able to relieve low back pain [18].

During lifting, it is well known that the main part of the axial loading of the spine is due to the large muscle and ligament forces applied over small internal lever arms. Importantly, it is thought that the amount of lumbar flexion (reduction in lordosis) determines the amount of ligamentous involvement in internal loading generation [19,20], which may or may not be present during heavy lifts [21]. Due to the smaller lever arms of the ligaments compared to the muscles, preserving sufficient lordosis when lifting can reduce the bone-on-bone loading between the vertebral bodies due to lower posterior ligament tension [21]. However, the preservation of 1-3° margin from full lumbar flexion seems to be sufficient to avoid overloading, and this is consistent with the kinematics observed in highly skilled lifters [21].

GMs and DLs are comparable in their ability to train agility, speed and power in all sport types [22], including typical strength exercises for ACL rehabilitation [23], but also for potential injury risk during exercising [24]. Despite the widespread use of GMs and DLs, the critical differences in lower limb and trunk motion, and more importantly the resulting loading conditions on the joints, during GMs and DLs remain unknown. This study therefore aimed to compare the segment kinematics and joint moments of the lower limbs and spine during the entire lifting action and at the point of deepest flexion during GMs and DLs in the sagittal plane.

## Methods

Nine male and four female subjects with experience in weight training (average age 24.5 ± 4.3 years, mass 74 ± 11 kg, height 180 ± 7 cm) were analysed while performing DLs and GMs exercises. The study was approved by the Ethics committee of ETH Zurich, Switzerland (EK 2012-N-57). One subject provided written informed consent to the publication of their images and all subjects provided written informed consent to participate in the study.

**Figure 1:**
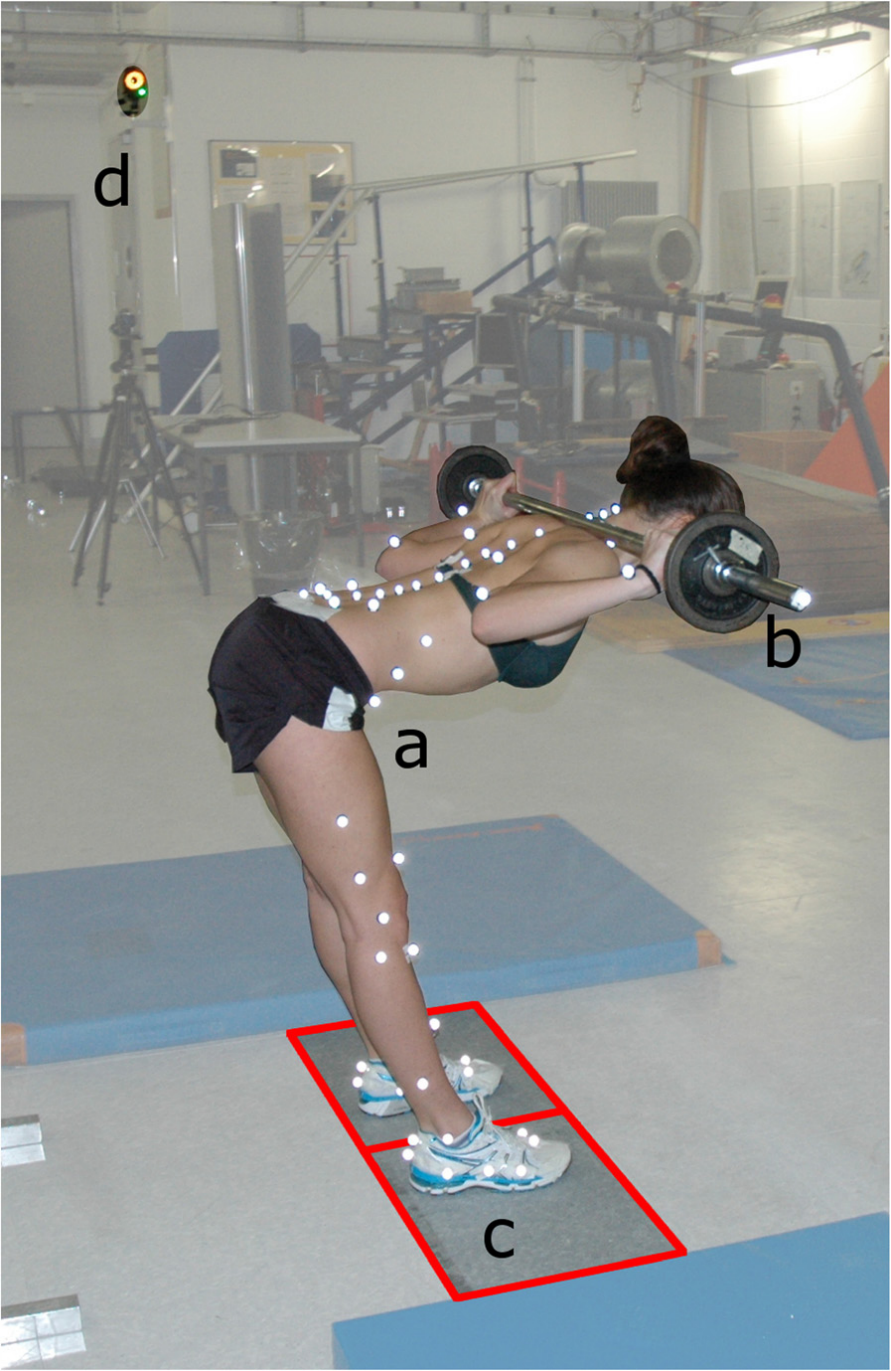
**Measurement set up including the following: a)** subject with the IfB Marker Set, **b)** barbell, **c)** force plates under each foot and **d)** 1 of the 12 Vicon cameras.

To analyse the motion of the body, an opto-electronic system (Vicon, Oxford Metrics Group, UK) with twelve cameras (MX40) and a sampling frequency of 100 Hz was used. The ground reaction forces were measured using two 400 × 600 mm force plates (type 9281B Kistler, Winterthur Switzerland), one under each foot, with a frequency of 2 kHz. The IfB Marker Set [25], consisting of 55 markers on the legs, pelvis, shoulder and arms, 22 on the back and 2 attached to the barbell, was used (Figure 1). The markers near the spine and on the rear and forefoot had a diameter of 9 mm while 14 mm markers were used on all other segments. Each marker was attached after palpation using double sided skin friendly tape by trained personnel.

Subjects wore their normal shoes for fitness training and shorts, while females additionally wore a bikini top. After a gentle warm-up session running on a treadmill or lifting an unloaded bar, the subjects’ performed the basic motion tasks [25]. The subject received standardized instructions for the two exercises (See section Standardised instructions for DLs and GMs). Both exercises started in the upright position. For the GMs, the subjects were advised not to bend their knees. A set of GMs with eight repetitions and an extra load of 25% body weight (BW) on the bar was then performed with the bar positioned on the upper trapezius muscle. Afterwards, the subjects additionally performed sets of eight repetitions each for DLs with 25% and 50% BW on the bar, with the extra loads representing a typical loading of a healthy non-powerlifters and was normalised to percentage of BW.

### Standardised instructions for DLs and GMs

General instructions:

1. Stand upright with your feet approximately shoulder width apart.

2. Point the feet slightly outward, following the natural divergence of the feet.

3. Lift the thorax to a natural spine position.

4. Hold tension in the core muscles during execution of the exercises.

5. Breathe out during the ascent.

6. Perform the exercise at the same normal speed during the downward and upward movements.

7. Lowest point before turn: No flexion in the lumbar spine.

DL specific instructions:

1. Hold the barbell with a comfortable grip, one hand in a supinated and the other in a pronated position.

2. Keep the head in a horizontal view.

GM specific instructions:

1. Put the barbell on the rear musculus deltoideus and hold it in a comfortable hand position.

2. Keep the head in extension of the spine.

The motion data were reconstructed in Vicon Nexus (version 1.7.1, Oxford Metrics Group, UK). The definition of a repetition of both of the two exercises was based on the start and stop point by using the vertical velocity of the two markers attached on the barbell (v_barbell_ > 0.04 m/s). The repetitions were time normalized and averaged. In addition, the maximum value for each repetition was averaged. Additionally, the force plates were specifically calibrated to allow for correction of the centre of pressure (COP) [26] and hence maintain accuracy during the inverse approach. The joint centres of the knee and hip were functionally determined from the basic motion tasks [25], and the joint centre of L4/L5 was defined anatomically based on anthropometric data [27]. The external joint moments in the sagittal plane were calculated using an inverse approach with a quasi-static solution [28], taking the ground reaction force and kinematic data into account [29], and normalized to BW [30]. The flexion / extension moments at the knees and hips were averaged over both limbs. The inverse approach included the position of the joints, the forces acting on each foot, and the gravitational force of the segments [30]. Due to slow accelerations of the segments during these exercises, the inertia forces were neglected. All calculations were performed in Matlab (version 8, The MathWorks Inc., Natick, MA, USA).

The position and orientation of each segment was determined relative to the reference segments defined by the standing trial as the neutral position (0° rotation) using a least-squares fit of the corresponding marker point clouds [25]. Joint rotations were described using a helical axis approach and for clinically interpretable rotational components, the attitude vectors were decomposed along the axes of a segment fixed, orthogonal, anatomically defined joint coordinate system [25]. The joint angles, the curvature and the moments were all analysed in the sagittal plane.

The influences of the extra barbell load (25% BW and 50% BW) and the type of lift (DLs and GMs) on the maximal segmental angles of the knee and hip, on the maximal and minimal segmental angle of the pelvis relative to lumbar spine and lumbar spine relative thoracic region, on the corresponding ranges of motion (RoMs) of the knee, hip, lumbar and thoracic spines (segmental and curvature approach) as well as on the normalized maximal moments of the knee, hip and L4/L5 were analysed using a multiple repeated-measures ANOVA (with significance defined at p < 0.05). Bonferroni adjustment, for the three groups, as well as for the number of parameters, was then conducted to establish significant differences, resulting in significance defined at p < 0.0033. All eight repetitions of every subject were averaged for the statistical analyses. Statistical calculations were performed using IBM SPSS software (version 21, SPSS AG, Zurich, Switzerland).

## Results and discussion

### Knee and hip

#### Kinematics

The observed maximal knee and hip rotations in the sagittal plane, as well as their RoMs, were significantly smaller during GMs than during DLs (Table 1). The typical observed intrasubject standard deviation over eight repetitions of the maximal joint angles of the knee and hip as well as of their RoM was < 2.5°. These results in the knee were expected due to the type of lifting execution during GMs, where the knee remains almost straight. The smaller flexion movement of the hip during GMs could be a restriction due to the limited length of the two-joint hamstring muscles in the extended knee position. The obtained maximal knee angles during DL were slightly larger than those observed by Brown EW and Abani K [31], while the hip angles remained comparable. No changes in the maximal knee and hip angles or their corresponding RoMs were found between the loading conditions with 25% and 50% BW during DLs. The observed RoMs during DLs of the knee and hip were in agreement with those observed in the study by McGuigan MR and Wilson BD [9].

**Table 1:** **Maximal segmental flexion angle of this and other studies**[1,6,9,31]**, range of motion (RoM) of knee, hip, pelvic-lumbar and lumbar-thoracic rotations (in degree) in the sagittal plane as well as the RoM of the curvatures (1/m) of the lumbar and the thoracic spine**

				**This study**		**Brown, 1985**		**Escamilla, 2001**	**Escamilla, 2000**	**McGuigan, 1996**
			**GM (25%)**	**DL (25%)**	**DL (50%)**	**DL (269%)**	**DL (197%)**	**DL (181%)**	**DL (289%)**	**DL (254%)**
Maximal segmental flexion angle	knee [°]	Mean	5.3	107.8*	103.4*	57.5	69.2	59.0	56.0	60.0
		SD	6.7	22.4	22.6	5.7	8.2	12.0	9.0	10.0
	hip [°]	Mean	75.3	103.2*	101.7*	110.9	115.9	124.0	108.0	113.0
		SD	9.2	4.8	6.0	5.1	5.0	8.0	21.0	5.0
	pelvis-lumbar [°]	Mean	23.8	27.4	25.7					
		SD	6.3	5.4	4.7					
	lumbar-thoracic [°]	Mean	0.9	2.3	5.0					
		SD	8.9	7.9	9.1					
Min segmental flexion angle	pelvis-lumbar [°]	Mean	7.0	6.4	6.8					
		SD	3.1	2.2	2.3					
	lumbar-thoracic [°]	Mean	−8.1	−4.7	−3.2					
		SD	7.5	7.3	8.2					
ROM segmental sagittal-plane rotations	knee [°]	Mean	7.8	103.4*	99.7*					
		SD	5.5	23.1	21.7					
	hip [°]	Mean	58.4	90.4*	89.1*					
		SD	10.0	5.3	5.9					
	pelvis-lumbar [°]	Mean	16.8	21.1	18.9					
		SD	4.7	4.3	3.5					
	lumbar-thoracic [°]	Mean	8.9	7.1	8.1					
		SD	3.8	2.8	3.5					
ROM of the curvature of the spine	lumbar [1/m]	Mean	2.7	2.7	2.5					
		SD	1.3	1.2	0.9					
	thoracic [1/m]	Mean	0.9	0.6	0.6					
		SD	0.6	0.3	0.3					

*: significantly different from GM.

#### Kinetics

During DLs, no changes between the two loading conditions (25% / 50% BW) on the barbell were found in the maximum moment about the knee in the sagittal plane. This seems rather surprising; one might expect higher loading due to the additional load on the bar (Table 2). However, it seems that a slight change of the trunk position is able to considerably modify the sagittal moments about the knee and therefore negate the effect of the extra load. In doing so, the subjects have managed to avoid additional loading at the knees. On the other hand, the hip flexion moment, as expected, increased significantly with additional weight on the barbell during DLs (Table 2).

**Table 2:** Mean normalized moments and standard deviations (SD) (N*m/BW) in the sagittal plane about the knee, hip and L4/L5 region for the GM with 25% extra load, DL with 25% and 50% extra load, respectively and corresponding results from other studies[1,6,12,15,31]

		**This study**			**Burnett, 2002 $**		**Escamilla, 2001 $**	**Escamilla, 2000 $**	**Cholewicki, 1991 $**	**Brown, 1985, $**	**Brown, 1985, $**
**Moment**		GM (25%) [N*m/BW]	DL (25%) [N*m/BW]	DL (50%) [N*m/BW]	GM (72%) [N*m/BW]	RDL (133%) [N*m/BW]	DL (181%) at LO [N*m/BW]	DL (289%) at LO [N*m/BW]	DL (259%) [N*m/BW]	DL (269%) at LO [N*m/BW]	DL (197%) at LO [N*m/BW]
Knee	Mean	−0.96	1.11*	1.14*	-	-	0.72	2.19	0.11	0.28	0.03
	SD	0.21	0.39	0.45			1.02	1.91	0.08	-	-
Hip	Mean	1.63	1.40*	1.92*^+^	-	-	4.86	7.80	4.44	2.76	2.18
	SD	0.14	0.13	0.19			1.79	2.99	0.17	-	-
L4/L5	Mean	2.75	2.81	3.77*^+^	3.78	3.63			7.78	-	-
	SD	0.26	0.27	0.43	0.72	0.97	-	-	0.25		

*: significantly different from GM (25%).

+: DL (50%) significantly different from DL (25%).

RDL: Romanian deadlift.

LO: Lift off.

$: normalized to body weight and divided by 2 for the knee and hip.

**Figure 2:**
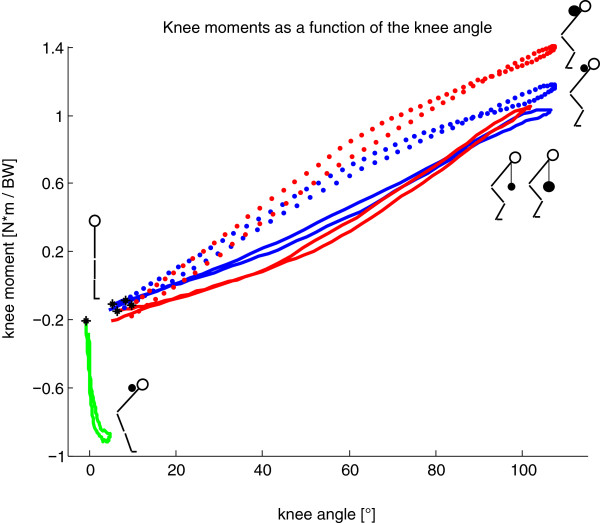
Normalized knee moments in the sagittal plane [N*m/BW] (positive for external knee flexion moment) averaged over all repetitions for all subjects during Goodmornings and Deadlifts with corresponding knee flexion angle ([°], zero represents a straight leg, defined on the basis of the standing trial) compared to the squat exercise (data taken from [30]**).** *: Starting point of the eccentric phase at the upright position; blue: DL with 25% extra load; red: DL with 50% extra load; green: GM with 25% extra load; blue dotted: squats with 25% extra load; red dotted: squats with 50% extra load.

During GMs, an external extension moment acted at the knee, while during DLs, from a knee flexion angle of 25° and higher, the moment produced was a flexion moment (Figure 2). The maximum external knee moments during DLs (Table 2) were comparable to knee moments in the studies of Escamilla and co-workers [1,6], although they used much higher barbell loads. Contrary to this finding, the studies of Cholewicki J, McGill SM and Norman RW [12] and Brown EW and Abani K [31] showed slightly lower knee moments in comparison to the present study (Table 2).

With the same extra load (25% BW), the sagittal moments about the hip were significantly larger during GMs compared to DLs (Table 2). However, the sagittal hip moments calculated for the current study were 2 to 6 times smaller than the aforementioned studies [1,6,12,31] (Table 2), but this is in line with the reduced barbell loading and was therefore entirely expected.

**Figure 3:**
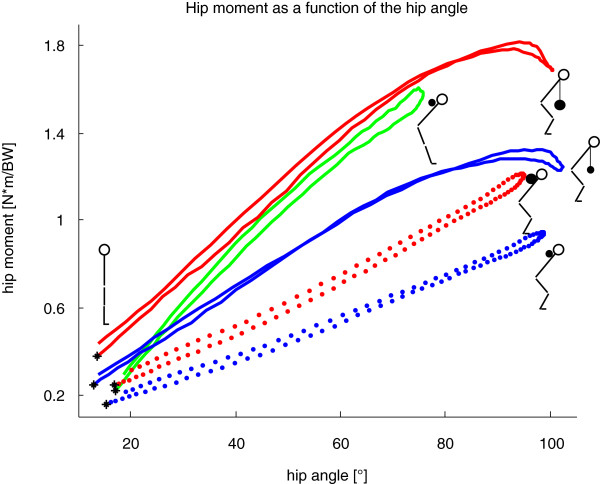
Normalized hip moment in the sagittal plane [N*m/BW] averaged over all repetitions and all subjects (positive for external hip flexion moment) with corresponding hip flexion angle ([°], zero represents a straight hip, defined on the basis of the standing trial) compared to the squat exercise (data taken from [30]**).** *: starting point of the eccentric phase at the upright position; blue: DL with 25% extra load; red: DL with 50% extra load; green: GM with 25% extra load; blue dotted: squats with 25% extra load; red dotted: squats with 50% extra load.

The largest extension moment at the knee was, in fact, observed during GMs (Table 2). It should be noted that GMs are a rather isometric exercise for the knee flexors, but the hamstrings undergo eccentric and concentric contraction due to motion at the hip (Figure 2). At the same extra weight, the RoM of the hip throughout the exercise was significantly smaller but the hip sagittal moment significantly larger during GMs compared to DLs (Figure 3). The largest RoMs and the highest sagittal moment in the hip were observed during DLs with 50% extra load (Figure 3).

The motion and loading patterns of DLs were observed to be similar to squats [30]. Here, the maximum knee flexion angle was within a few percent (<4%) whereas the maximal flexion moment in the knee was higher for squats with 50% extra load (Figure 2). Larger differences were observed in the hip, however, where the maximal flexion moment during DLs was at least 50% larger (Figure 3).

#### Relevant outcomes for ACL injury prevention

A number of studies have identified the force ratio of the quadriceps to hamstring (H:Q) as a risk factor for ACL rupture [32], especially in women [33]. Holcomb and co-workers [33] used, amongst other exercises, GMs and straight leg DLs to modify the H:Q ratio by training within 6 weeks. For the training of multi-joint muscles, such as the M. semimembranosus and semitendinosus, the joint angles of the hip and the knee, as well as their corresponding moments, should be taken into account. Based on the finding that maximal external extension moment in the knee (Figure 2) and a flexion moment in the hip were observed during GMs (Figure 3), it follows that GMs might provide an effective strategy for focused strengthening of the hamstrings. This finding is in agreement with the study of Ebben W [32], who demonstrated the importance of hamstring training for the potential reduction of ACL injuries and who further recommended GM training to be included as a preventative measure.

One new and notable outcome of this study is that no larger external knee moments were observed by a larger extra load on the barbell during DLs (Table 2). Compared to squats [30], DLs have the advantage that the flexion moment in the knee is smaller (Figure 2) but the flexion moment in the hip is larger (Figure 3) using the same extra load. Therefore, based on the observed kinetics and kinematics of the strength exercises, the following ranking is suggested in order to shift the H:Q ratio towards H: GM, DL 50%, DL 25% and squats.

### Back

#### Kinematics

Neither the RoMs of the lumbar and the thoracic curvatures, nor the maximal and minimal flexion and extension angles of the pelvic-lumbar and the lumbar-thoracic segments, were affected by the execution or the extra weight on the barbell (Table 1).

#### Kinetics

The flexion moment in the L4/L5 region was significantly higher during DLs than GMs due to the additional load on the barbell (Table 2). However, the two exercises produce the same loading conditions at the L4/L5 region using the same load on the barbell (Table 2). Previously presented data of normalized moments in L4/L5 using 259% BW load during conventional DLs [12] were larger compared to the values of the present study (Table 2). However, the study from Burnett A, Beard A and Netto K [15] showed smaller moments at L4/L5 during Romanian deadlift exercises compared to the present study, even though they used much higher normalized weights on the barbell (133% BW extra load). During GMs, Burnett and co-workers [15] found higher moments at L4/L5 compared to the present study, which could be explained by the heavier extra loading on the barbell (72% BW extra load).

#### Back training

**Figure 4:**
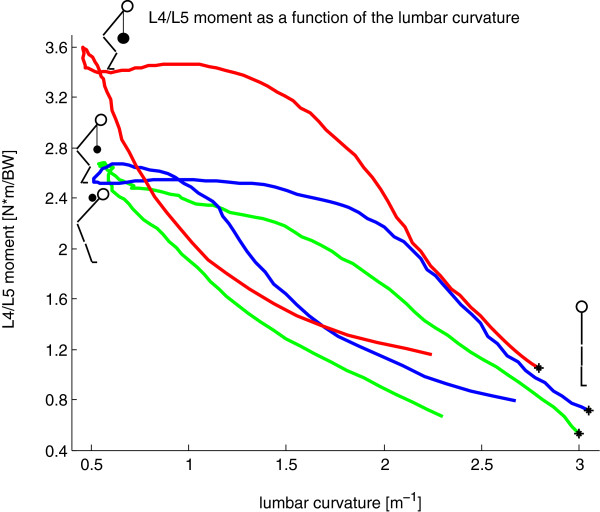
Normalized L4/L5 moment in the sagittal plane [N*m/BW] averaged over all repetitions and all subjects (positive for external L4/L5 flexion moment) with corresponding lumbar curvature [1/m]. *: starting point of the eccentric phase at the upright position; blue: DL with 25% extra load; red: DL with 50% extra load; green: GM with 25% extra load.

Surprisingly, the relationship between the L4/L5 moment and the lumbar curvature in the sagittal plane was different between the concentric and the eccentric phases of lifting; especially during DLs using 50% extra weight (Figure 4). During the eccentric phase, the lumbar back maintained its higher curvature longer compared to the concentric phase with the same sagittal moment (Figure 4). Due to the fact that the flexion moments at L4/L5 were similar for the two exercises with 25% BW extra load (Table 2) and no differences in the RoM of curvature or the segmental kinematics of the back were observed (Table 1), from a biomechanical point of view, the two execution types are comparable for the trunk. As a result, differences between the exercises of the kinematics and mechanics in the lower limbs should be considered more relevant.

### Practical applications

In order to optimize the training effect of the quadriceps, a large RoM [34] and external flexion moment in the knee is demanded. It follows that the DL is the preferable exercise for quadriceps training, although the extra load did not affect the sagittal moment in the knee. During squatting, similar RoMs in the knee, but higher moments in the sagittal plane and a load dependency of the moments have been observed [30].

To train the M. gluteus maximus, GMs produce a higher sagittal moment but a smaller range of motion than DLs. If a large range of motion is required, DLs are therefore considered the better choice. The small RoM in the knee suggests that GMs should be chosen before DLs at the early stage of rehabilitation for subjects with a previous knee injury. Furthermore, GMs are suited to avoid external flexion moments at the knee. The magnitude of the resulting extension moments during GM is similar to the magnitude of the flexion moment during DLs.

## Conclusions

DLs and GMs show different motion and loading patterns for the lower extremities, where the knee remains almost straight during GMs, hence producing a large extension moment. The maximal knee and hip angle, as well as the RoMs of the knee and hip, are smaller during GMs than DLs. Kinematically, the DL is not generally affected by the extra weight on the barbell. The flexion moment at the knee during DLs is also not influenced by the additional 25% load, however, the sagittal moment in the hip is higher during DLs using 50% BW extra load. Based on the higher sagittal moments in the hip and the L4/L5 region with higher barbell loads, great care should be taken to ensure core stability of the trunk during lifting due to high loading of the spine, especially when training with higher extra loads. Finally, for prevention of ACL injuries, GM are recommended for training the hamstrings to quadriceps ratio.

## Competing interests

The authors declare that they have no competing interests.

## Authors’ contributions

FS and JL acquired the data, and undertook the data analyses including the preparation of the tables and figures. FS furthermore performed the statistical analysis and helped drafting the manuscript. RL supported the measurement set up, data analysis and helped drafting the manuscript. BT helped in both interpreting the data and drafting the manuscript. SL made the concept and design of this study, supervised the data analyses and interpretation and helped drafting the manuscript. All authors read and approved the final manuscript.

## Pre-publication history

The pre-publication history for this paper can be accessed here:

http://www.biomedcentral.com/2052-1847/5/27/prepub
